# 2288. Short and Longer-Term All-Cause Mortality among SARS CoV-2-Infected Persons and the Pull-Forward Phenomenon in Qatar

**DOI:** 10.1093/ofid/ofad500.1910

**Published:** 2023-11-27

**Authors:** Hiam Chemaitelly, Jeremy Samuel Faust, Harlan M Krumholz, Houssein H Ayoub, Laith J Abu-Raddad

**Affiliations:** Weill Cornell Medicine - Qatar, Doha, Ad Dawhah, Qatar; Brigham and Women’s Hospital, Boston, Massachusetts; Yale University School of Medicine, New Haven, Connecticut; Weill Cornell Medicine - Qatar, Doha, Ad Dawhah, Qatar; Weill Cornell Medicine-Qatar, Doha, Ar Rayyan, Qatar

## Abstract

**Background:**

Risk of short- and long-term all-cause mortality after a primary SARS-CoV-2 infection is inadequately understood.

**Methods:**

A national, matched, retrospective cohort study was conducted in Qatar to assess the risk of all-cause mortality in the national cohort of people infected with SARS-CoV-2 compared with a reference national control cohort of uninfected persons. Associations were estimated using Cox proportional-hazards regression models.

**Results:**

Among unvaccinated persons, within 90 days after primary infection, adjusted hazard ratio (aHR) comparing incidence of death in the primary-infection cohort with the infection-naïve cohort was 1.19 (95% CI: 1.02-1.39). The aHR was 1.34 (95% CI: 1.11-1.63) in persons more clinically vulnerable to severe COVID-19 and 0.94 (95% CI: 0.72-1.24) in those less clinically vulnerable to severe COVID-19. In subsequent follow-up, the aHR was 0.50 (95% CI: 0.37-0.68). The aHR was 0.41 (95% CI: 0.28-0.58) in months 3-7 after the primary infection and 0.76 (95% CI: 0.46-1.26) in subsequent months. The aHR was 0.37 (95% CI: 0.25-0.54) in persons more clinically vulnerable to severe COVID-19 and 0.77 (95% CI: 0.48-1.24) in those less clinically vulnerable to severe COVID-19. Among vaccinated persons, no evidence was found for differences in incidence of death in the primary-infection versus infection-naïve cohorts, even among persons more clinically vulnerable to severe COVID-19.

**Conclusion:**

COVID-19 mortality in Qatar appears primarily driven by forward displacement of deaths of individuals with relatively short life expectancy and more clinically vulnerable to severe COVID-19. Vaccination negated the mortality displacement by preventing early deaths.

**Disclosures:**

**All Authors**: No reported disclosures

